# Incorporating patients’ input in the development and validation of patient-reported outcome measures in oncology: a comprehensive scoping review

**DOI:** 10.1186/s12955-026-02500-4

**Published:** 2026-02-18

**Authors:** Iryna Shakhnenko, Shushan Hovsepyan, Emily I. Holthuis, Helen Bulbeck, Winette T. A. van der Graaf, Olga Husson

**Affiliations:** 1 European Organization for Research and Treatment of Cancer Headquarters, Brussels, Belgium; 2 Department of Medical Oncology, The Netherlands Cancer Institute, Antoni van Leeuwenhoek, Amsterdam, Netherlands; 3 Brainstrust, Cowes, Isle of Wight, UK; 4 Department of Medical Oncology, Erasmus MC Cancer Institute, Erasmus University Medical Centre, Rotterdam, Netherlands; 5 Department of Surgical Oncology, Erasmus MC Cancer Institute, Erasmus University Medical Centre, Rotterdam, Netherlands; 6 Departments of Public Health, Erasmus MC Cancer Institute, Erasmus University Medical Center, Rotterdam, The Netherlands

**Keywords:** Patient involvement, Patient participation, Patient-reported outcome, PROM, Clinical research, Oncology, Cancer, Item development, Validation

## Abstract

**Background:**

Patient-reported outcome measures (PROMs) play a crucial role in evaluating oncological interventions. Although PROMs are developed to assess the patient’s health status, it remains unknown if patients’ perspectives are considered when developing and validating PROMs. This review aims to describe the nature of patient involvement in PROM development and validation in oncology research.

**Methods:**

A scoping review was conducted to identify relevant articles on patient involvement in PROM development and validation in cancer research published between 1993 and 2023 using PubMed/Medline, Embase, Scopus, and Cochrane databases, with the aim of exploring the extent and nature of patient involvement in developing and validating PROMs specifically in cancer research.

**Results:**

Out of 1354 initially identified articles, 34 met the inclusion criteria, and included various cancer types. The majority of studies (76%) engaged patients in item development through methods such as focus groups and interviews. Patient involvement across all phases of development and validation was documented in 35% of the studies. The value of patient involvement was illustrated by the improved relevance of the PROM content, leading to the inclusion of issues that were meaningful to patients.

**Conclusion:**

This scoping review demonstrates that even though the nature and extent of involvement varied throughout the stages of PROM development, the value of patients’ voices was not in question. A potential next step could be to introduce standardized terminology and develop guidelines to enhance a consistent and effective integration of patients’ input into the development of PROMs in the oncology setting.

**Supplementary Information:**

The online version contains supplementary material available at 10.1186/s12955-026-02500-4.

## Background

A patient-reported outcome (PRO) refers to direct patient-derived information about their health condition, and it constitutes an indispensable element of healthcare when evaluating interventions [1, 2]. Patient-reported outcome measures (PROMs) are instruments to measure PROs and include self-reported questionnaires or surveys [2–4]. While historically PROMs were designed to assess treatment safety in clinical trials, today they are used in clinical research as co-primary or secondary endpoints to determine the net clinical benefit of treatment [3, 4]. Besides objective outcomes, which include among others response and survival, PROs provide additional information related to various health-related constructs, such as health-related quality of life (HRQoL), functional status, and the manifestation and burden of symptoms reported by the patient [5]. One of the most assessed PROs is HRQoL. It represents a multidomain concept that refers to the subjective evaluation of one’s overall perception of the impact of an illness and its treatment, on physical, psychological, and social well-being [6].

Medical and pharmaceutical organizations, regulatory agencies, and health insurance providers encourage physicians and researchers to apply PROMs in clinical trials according to available international standards such as SISAQOL (Setting International Standards in Analyzing Patient-Reported Outcomes and Quality of Life Endpoints Data) and Food and Drug Administration’s Guidance for Industry [2, 7–12]. There are several guidelines available for the development of PROMs, with most of them focusing on qualitative and quantitative research methods to create high-quality PROMs. For instance, COSMINE guidance outlines structured approaches for incorporating patient input to evaluate the content validity of PROMs, including assessment of item relevance, comprehensiveness, and comprehensibility [13].

In oncology, the guidelines developed by the Quality of Life Group of the European Organisation for Research and Treatment of Cancer (EORTC) are most often applied, describing four development stages: (1) identifying the pertinent quality of life issues, (2) transforming these issues into a set of items, (3) conducting preliminary testing of the item list in the questionnaire, and (4) undertaking extensive field testing on an international scale [14]. However, these guidelines do not describe how to incorporate the patient’s perspective in these phases.

Nowadays, there is an increasing recognition of patients’ valuable experiential knowledge as a crucial information source for enhancing research. Patient involvement refers to patients and caregivers contributing their perspectives to the research as distinct from the term “participation”, when patients take part in research as participants [15]. The growing consensus of actively involving patients in developing, validating – where patients indicate that items cover important aspects of their experiences [16] – or implementing PROs is not only an ethical call but also highly beneficial in the context of research, by enriching discussions and introducing novel items that could contribute to the tools’ validity [3]. Patient involvement offers first-hand perspectives of the patients on the disease, assists in identifying relevant endpoints, and ensures that PRO assessments reflect patient experiences, including adverse events [9, 19–21].

Organizations like INVOLVE in the UK, Patient-Centered Outcomes Research Institute (PCORI) in the USA, and the Canadian Institutes of Health Research advocate for the active involvement of patients and encourage patients to act as partners, contributing to research planning and decision-making [17–19]. Input provided by patients at various stages cultivates a mutually advantageous collaboration between patients, patient representatives, caregivers, and medical professionals [20, 21].

Most studies dedicated to patient involvement in developing and/or validating PROMs were conducted in fields other than oncology, such as chronic diseases, diabetes, obesity, etc. [22–25]. A noteworthy example in oncology, specifically in breast reconstruction, demonstrated the benefits of active patient involvement where patients played a significant role in various aspects of the study, including co-defining which outcomes to measure, participating in interviews for item development, and contributing to the testing for comprehensibility of PRO instruments [26]. However, despite the growing importance of involving patients from the beginning of the PROM development, generally, PROMs are still created without patients’ input [3]. For the optimal integration of the patient perspective into the entire PROM development, implementation, and dissemination process, its inclusion in the initial stages is essential [8]. Our review aims to thoroughly explore the extent and nature of patient involvement in developing and validating PROMs specifically in cancer research building on Wiering et al.‘s broader evaluation of patient involvement in PROMs development in general healthcare.

## Main body

### Methods

We performed a scoping review following the PRISMA guidelines [27, 28]. The databases PubMed/Medline, Embase, Scopus, and Cochrane were searched throughout September 2023 and identified articles published between 1993 and 2023 using three major Mesh terms; “patient involvement” AND “patient-reported outcomes” AND “neoplasms” (Appendix 1). The search strategy was developed with input from an experienced librarian to ensure the precision and thoroughness of our selection of the key terms. Additionally, recent literature reviews were scanned for relevant citations. All retrieved citations were exported to Mendeley Cite and duplicates were removed. Inclusion and exclusion criteria were defined before the search. Search results were imported into the web-based tool Rayyan to facilitate study screening.

Three authors independently screened the titles and abstracts of all retrieved citations and, further, they reviewed full texts of the remaining articles using the review’s eligibility criteria. Reviewers were not blinded to each other’s decision; disagreements were resolved through discussion. Basic information was extracted from the articles, such as first author, year of publication, country, keywords, summary points (key findings and relevance to patient involvement in PROM development), age group, PROM development, validation phase, and nature of patient involvement.

Additionally, we included Helen Bulbeck as a patient partner in this project. Her input guided us throughout this review and shed light on the need not only to clarify the terms “involvement” and “participation”, but also to elaborate on the difference between “involvement” and “engagement”.

#### Inclusion criteria

Studies were included for full-text review if they met at least one of the three criteria: (i) a description of PROM development with the help of patients (different phases of development, such as outcome, item, checking the questionnaire, etc.), (ii) a description of PROM validation with the help of patients; (iii) studies that detailed the collaboration, or engagement in the processes of PROM development and/or validation (Table 1). We relied on the author’s understanding of the terminology of “participation” and “involvement,” however, these terms were occasionally used interchangeably requiring careful interpretation to maintain consistency with our analytical framework.

We are aware that “patient engagement” refers to disseminating information and knowledge about research with the lay audience. However, in the scope of this research, our focus lied on a more active role of patients in the PROM development. For this reason, we will further explore only the concept of “patient involvement”.

**Table 1:** PCC inclusion criteria for scoping review

Population	Patients who have actively participated in the development or validation of PROMs in cancer research
Concept	Patient involvement in PRO development and/or validation process in cancer research
Context	Studies in cancer research involving patients/patient representatives in PRO development, validation

PCC: Population, Concept, Context; PROMs: patient-reported outcome measure; PRO: patient-reported outcome

#### Exclusion criteria

Exclusion criteria included articles that did not directly discuss patient involvement in PROM development, validation, or selection of PROM endpoints in cancer research. Studies not involving cancer patients and Non-English language publications were also excluded.

#### Data extraction

The citation, title, summary points, and DOI of the articles identified during the hand search were captured in an Excel spreadsheet and added to the literature search results for screening.

## Results

### Search flow

**Fig. 1:**
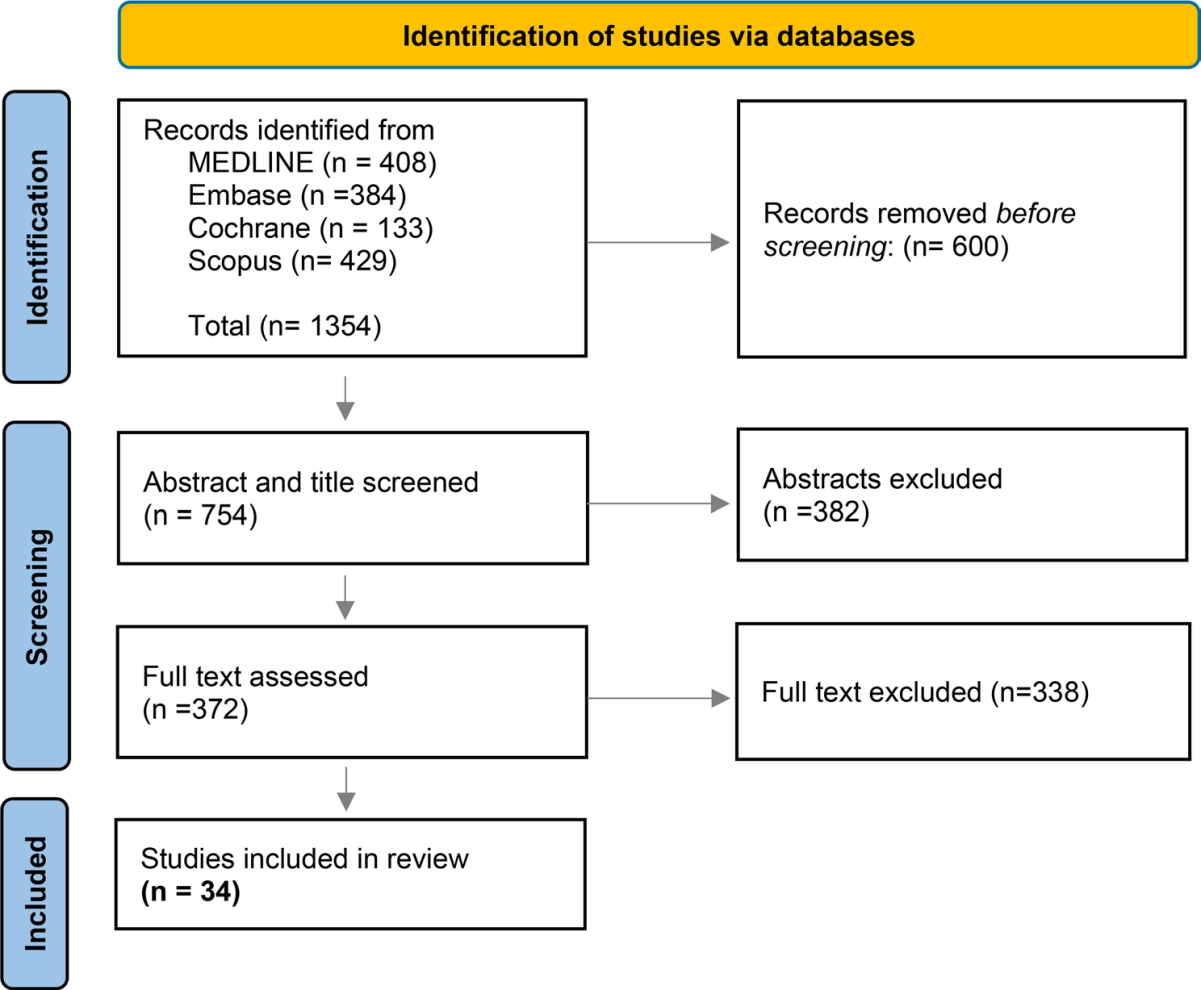
Identification of studies via databases

Figure 1 shows the flowchart of articles through the selection process. The initial search of the PubMed, MEDLINE, Embase, Scopus, and Cochrane peer-reviewed literature databases resulted in 1354 articles. After removing duplicates (*n* = 600), the titles and abstracts of 754 articles were screened for eligibility. Of these, 382 articles were excluded because they were not cancer-specific or did not involve patients in the PROM development, validation, or implementation phase. The remaining 372 underwent full-text screening. A final set of 34 articles was included, with agreement reached by three authors.

### Study characteristics

Out of 1354 articles, 34 studies described patient involvement in the PROM development and/or validation phases. The baseline characteristics of the included studies are summarized in Table 2. The studies included in this scoping review were published between 1993 and 2023.

From a geographical perspective, the majority of the studies were carried out in the United States (*n* = 13) and in the United Kingdom (*n* = 7). Additionally, studies were conducted in Canada (*n* = 4), The Netherlands (*n* = 4), Italy (*n* = 2), and one each in Greece, Norway, Switzerland, and Denmark. The studies with patient involvement in the PROM development/validation covered diverse types of cancer: head and neck cancers, skin cancer, colorectal cancer, pancreatic cancer, prostate cancer, breast cancer, and multiple myeloma. The evaluated outcomes were mainly HRQoL, as well as disease- and treatment-specific outcomes, such as xerostomia after parotid-sparing surgery or swallow outcomes after laryngectomy for head and neck cancers.

### Patient involvement

In the process of PROM development and validation, patients were actively engaged through various approaches. Across the 34 studies, 76% (*n* = 26) described patient involvement in the item development (generation, reduction, concept elicitation) process. The methods to consult patients’ input mainly included focus groups, semi-structured open-ended interviews or structured interviews. The interviews were conducted mostly in person. In some cases, patients were involved in pilot testing (44%), and module development (9%). In 35%, patients were involved in all phases of PROM development. Patients involved in the studies were individuals actively undergoing cancer treatment (82%) at the time of their engagement and cancer survivors in follow-up (18%). Only a few studies briefly mentioned patient knowledge of or education about PROMs [29–32].

**Table 2:** Development and validation of PROMs: patient involvement

Stage	Activities	Number (%) of studies
Item development [16, 29, 30, 33–54]	Generation, reduction, concept elicitation, scale	26 (76%)
Module development [38, 54, 55]	Generation of QoL issues, construction of the item list	3 (9%)
Pilot testing [29–31, 33, 40, 41, 43, 45, 50, 51, 54, 56–59]	Pre-testing of questionnaires, content validation	15 (44%)
All phases of PROM development [16, 30, 32, 38, 40, 45,50, 54–56, 60, 61]	Item development and validation	12 (35%)

PROMs: patient-reported outcome measure; QoL: quality of life

Additional details regarding characteristics and methods of patient involvement are provided in Supplementary Table S1.

## Discussion

The findings showed a variation in the extent of patient involvement in PROM development studies, with over a quarter lacking any recorded involvement, potentially undermining the questionnaire’s alignment with patient perspectives and limiting its effectiveness in treatment decision-making. The studies stressed the need for consensus on patient involvement requirements and careful selection of PROMs based on the degree of patient involvement [3].

Considering the limited extent of patient involvement and the absence of established guidelines and recommendations for involving patients in the design and conduct of PROM development studies, this scoping review was undertaken with a specific focus on patient involvement in the development and/or validation of PROMs within the field of oncology.

Our findings emphasize the value and indispensability of involving patients in the development and validation of PROMs in cancer research. This is evidenced, among other examples, by the reduction of redundant items leading to the selection of the most important items in partnership with patients [46], as well as successful monitoring and management of cancer patients’ physical symptoms and psychological well-being [16, 29].

However, our findings also indicate variability in the nature and extent of patient involvement. Continuous patient involvement throughout all stages of PROM development was documented in a minority of studies [26, 62–66], with most studies reporting partial patient involvement. However, it’s important to highlight the challenges in maintaining consistent involvement throughout the lengthy development process, especially in a palliative care setting [67]. Transparent communication regarding the duration and scope of patient involvement is essential, recognizing that continuous involvement may not always be feasible or preferred, necessitating alternative strategies for meaningful input in PROM development [31].

In the prevailing cases, it was typical to observe a frequent use of the term “patient involvement” without an adequate description of the extent and nature of involvement. Moreover, the incorporation of the term “participation” within the broader context of “involvement” added complexity to the process of selecting eligible studies [26]. For example, Winters et al. described the process as patient participation, wherein patients assessed each item for “relevance” and “difficulty” on a four-point scale, followed by prioritizing the top five items. Additionally, a debriefing interview was conducted to evaluate item wording, ensure clarity, and address potential issues such as omissions, redundancies, or duplications demonstrating that these types of activities can be considered as “involvement” and not only “participation” [63].

Another example of interchangeable use of the terms “participation” and “involvement” is reported by Brunelli et al. stating “The adaptation of the questionnaire content to the specific patient population, will be performed with the contribution of clinicians of the multidisciplinary teams involved, *discussed with cancer patients’ representatives* and *pre-tested* among a restricted group of users” [51].

To address the potential confusion of terms, it is crucial to establish clear and distinct definitions that take into account the extent of patients’ input, their role in decision-making, and the degree of their active engagement [14, 26].

Some studies covered a diverse spectrum of cancer types, including colorectal, breast, and prostate (Supplementary Table S1), which inclusively allows to receive a wide range of patient perspectives. The questionnaires and surveys that were utilized in the item development process, typically require approximately 5 to 10 min to complete. They can be easily completed without assistance and readily integrated into the patients’ schedules [68, 69]. However, a skilled interviewer is required to establish good rapport and minimize the risk of participants getting side-tracked during the conversation, nevertheless, sometimes side-tracking can provide valuable findings. For instance, in semi-structured interviews, even though patients may deviate from the main topic, their input can still convey valuable information [70, 71].

Another interesting finding that goes in line with both FDA and the EORTC recommendations for the item generation [42], was the various number of patients involved. This underscores the importance of the quality of qualitative responses received, rather than focusing on the number of patients involved in the process [2, 14]. The authors interpreted this process as an instance of patient involvement; however, it might be more accurately categorized as patient participation rather than true involvement. In the context of involvement, patients and researchers would work together on the content and design of the questionnaire, interpretation of the results of patient interviews, and jointly define the next selection of items. In this way, the role of patients as study participants would transit to the role of patients as active collaborators in decision-making process.

The effectiveness of measurement scales in addressing disease and site-specific concerns, largely depends on their inclusivity of symptoms relevant to particular diseases, highlighting the need for tailored approaches in assessing PROMs [5]. In the study of Govender et al., patients were involved as experts in all phases of the development and preliminary validation of a questionnaire assessing swallow outcomes in patients with laryngectomees as it differs from other head and neck cancer PROMs. The patient volunteers who participated in the National Association of Laryngectomy Clubs (NALC) meeting were involved in group discussions for item generation. This meeting attendance could be considered indirect patient education for item development. Interestingly, some symptoms such as altered smell, self-consciousness while eating, and dry mouth were not identified by clinicians but were emphasized by patient focus groups as relevant factors to be included in the item list. This accentuates the critical role of the target population’s active involvement in the development of questionnaires [72].

Additionally, it is essential to adopt a multilingual and socioeconomic approach to maximize cross-cultural applicability [65]. As a case in point, Glaser et al. reported low involvement rates among the elderly or those living in low socioeconomic regions implying that people in these vulnerable categories may need to be evaluated using specific techniques [64]. For example, the group with a low participation rate will need specific education to be able to consult on the topics of item development and validation. To ensure that the intended target population will be actively involved, it is also important to gather input from diverse socioeconomic and cross-cultural groups to generate highly relevant items. These characteristics include, among others, ethnicity, gender, performance status, and treatment phase [14]. Patient advocacy organizations are available to offer relevant training and awareness sessions that could help tackle this issue and explain the benefits of PROs in research and clinical settings [8].

An alternative approach to assessing content validity could be the evaluation of web-based patient stories on social media. Patient-bloggers often provide comprehensive descriptions of their disease-related experiences in their blogs. In adherence to ethical principles, the bloggers were asked for consent (1) to utilize their posts and/or (2) relevant quotes with proper referencing, as a gesture of respect for their authorship rights. The comparison of the topics related to HRQoL in the breast cancer patients’ blogs revealed that the majority were identical to the BREAST-Q and the EORTC Core Quality of Life Questionnaire (EORTC QLQ-C30) [73]. In general, screening patient blogs for content validity can be seen as a non-standard approach to passive patient involvement. However, it is essential to rely on clear guidance and consensus in order to ensure the effectiveness and ethical adherence of such blogs.

Patient involvement in cancer clinical trials is influenced by different factors, such as the absence of standardized guidance, the subjective nature of PROs, and the challenges in translating patient’s input into practical applications [16]. It is challenging to synthesize the available data and evaluate the efficacy of various patient involvement strategies since there is no single, globally accepted definition of what constitutes involvement [17]. Moreover, additional efforts are required for the implementation phase, and it is imperative to provide a thorough description of the strategies employed in the studies.

To the best of our knowledge, no comprehensive, universal framework has been developed that outlines the effective way to involve patients and this is the first scoping review focusing on patient involvement in PROM development and/or validation within the field of oncology. This review not only provides direction for future research but also emphasizes the vital role of patients in shaping the relevance of PROMs in healthcare. Besides, it is important to note that the available literature tends to confuse patient involvement with patient participation. For instance, the EJP RD Short Guide on Patient Partnerships in Rare Disease Research Projects views “patient participation” not only as taking part in a research study as subjects/participants but also as an activity where patient representatives support recruitment [74]. We tend to disagree with the second part of this definition as it serves rather as an example of patient involvement.

Lastly, our findings should be interpreted not only as a call for standardized definitions and guidelines for patient involvement, but also the pressing need for more systematic and meaningful approaches to involving patients when developing PROMs in oncology setting.

### Limitations

This study has potential limitations. Even though patients’ input is being sought when developing PROMs in oncology, the distinction between patient participation and patient involvement appears to be rather vague. In this review, we have observed a variety of synonymous terms used to describe both phenomena – involvement and participation. Such terms include “patients’ perspective”, “patients voice”, “patients input”, “patients’ views”, and “patients’ beliefs”. The inability to properly differentiate patient participation – taking part in a study, from patient involvement – implying a partnership with patients and caregivers, might limit the ability of researchers to measure the extent of patient involvement in PROM development. This limitation suggests the need for a standardised approach to patients’ input collection in the quality-of-life setting.

Another limitation is that the search was conducted over two years ago. As patient involvement in PROM development is an evolving topic, more recent studies may be available.

## Conclusion

This review shows that patient involvement is commonly incorporated into the development and validation of PROMs in cancer research, most frequently during item generation and refinement. The findings highlight the important role patients play in shaping the relevance of PROM content. However, the review also revealed inconsistent reporting and a lack of clarity on what was regarded as patient involvement and at which stages of PROM development and validation it was considered essential. This underscores the need for clearer, standardized definitions and guidance to support meaningful and transparent patient involvement in PROM development.

## Supplementary Information

Below is the link to the electronic supplementary material.

Supplementary Material 1

## Data Availability

No datasets were generated or analysed during the current study.

## Declarations

### Approval committee

This project was approved by the Project and Module Development Committee and the Executive Committee of the EORTC Quality of Life Group.
