# Supplementary material for: Incorporating patients’ input in the development and validation of patient-reported outcome measures in oncology: a comprehensive scoping review
Source: Health Qual Life Outcomes. 2026 Feb 18;24:40. doi: 10.1186/s12955-026-02500-4 (PMC13020400; doi:10.1186/s12955-026-02500-4)
Supplement: Supplementary file 1 — Supplementary Material 1 [file 12955_2026_2500_MOESM1_ESM.docx]

**Supplementary Table S1. Characteristics of patient involvement**

| **Author, year** | **Domain measured** | **Cancer type** | **Methods of patient involvement** | **Stages of PROM development /validation** |
| --- | --- | --- | --- | --- |
| Cella, 1993, USA (1) | Health-related quality of Life | Cancer | Questionnaire | Item generation, item review/reduction, scale construction; Pilot testing, Initial and additional evaluation |
| Sprangers, 1996, The Netherlands (2) | Health-related quality of Life | Breast cancer | Interviews | Item development, pilot testing |
| Hearn, 1999, UK (3) | Physical, psychological and spiritual domains of life | Palliative care – various cancers | Questionnaire | Item development; Establishing the accuracy of the PROM |
| Eisbruch, 2001, USA (4) | Xerostomia, parotid-sparing irradiation | Head and neck cancer | Questionaries; cognitive interviews | Item development, content and construct validity |
| Vickery, 2001, USA (5) | Health-related quality of Life | Gastric cancer | Semi-structured interviews; cognitive interviews | Item development, construction of a provisional module; Establishing the accuracy |
| Mystakidou, 2004, Greece(6) | Health-related quality of Life | Palliative care – various cancers | Questionaries, structured interviews | Item development; Establishing the accuracy via scale analysis |
| Cano, 2005, UK (7) | Symptoms, psychological functioning, limitations of daily activities, cosmetic appearance and patient satisfaction | Head and neck skin cancer | Interviews, questionaries | Item development; item reduction, Establishing the accuracy via pretesting |
| Rhee, 2006, USA (8) | Health-related quality of Life | Non-melanoma skin cancer of head-and neck | Semi-structured interviews; questionnaires | Item validation -scale analyses |
| Rhee, 2006, USA (9) | Health-related quality of Life | Non-melanoma skin cancer of head- and neck | Semi-structured interviews, open- ended items, rating items in terms of their importance | Item generation and reduction |
| Rodrigues, 2007, Canada (10) | Health-related quality of Life (radiation toxicity) | Prostate cancer | Focus groups | Item generation; questionnaire development, Establishing the accuracy via questionnaire, pilot testing, item reduction; pilot-testing (partly) |
| Helbostad, 2009, Norway (11) | Physical functioning | Palliative care – various cancers | Questionnaire | Establishing the accuracy, pilot-testing (partly) |
| Wang, 2010, USA (12) | Gastrointestinal module | Gastrointestinal cancer | Qualitative and cognitive interviews | Item development, validation |
| Govender, 2012, UK (13) | Swallow Outcomes After Laryngectomy | Head and neck cancer | Focus group; cognitive interviews | Item development; Preliminary validation |
| Wagner, 2012, USA (14) | Health-related quality of Life | Multiple Myeloma | Quantitative item ratings | Item development, Establishing the accuracy based on qualitative research practices |
| Senn, 2012, Swizerland (15) | Postsurgical PROMs | Vulvar cancer | Interviews with open-ended questions | Item development; Pilot testing |
| Glaser, 2013, UK (16) | Health-related quality of Life | Breast, colorectal, prostate and non-Hodgkin's lymphoma | Cognitive interviews | Establishing the accuracy of the PROM |
| Flynn, 2013, USA (17) | Sexual Function and satisfaction | Various cancers | Focus groups, cognitive testing | Item development; Content validity and tool testing |
| Thomson, 2013, UK (18) | Health-related quality of Life | Breast cancer/reconstruction | Interviews; cognitive interviews | Item development; Establishing the accuracy |
| Winters, 2014, UK (19) | Health-related quality of Life | Breast cancer/reconstruction | Interviews; cognitive interviews | Item development; Establishing the accuracy |
| Mathias, 2014, USA (20) | Health-related quality of Life | Advanced basal cell carcinoma, basal cell carcinoma nevus syndrome | Telephone interviews; cognitive debriefing interviews | Item development, concept elicitation |
| Williams L, 2014, USA (21) | Health-related quality of Life | Ovarian cancer | Interviews | Item development; Subsequent module development in GI, lung, CML, cGVHD |
| Hay, 2014, USA (22) | PRO-CTCAE | Various cancers | Interviews | Content validity |
| Absolom, 2015, UK (23) | Health-related quality of Life | Various cancers | Interviews, via cognitive interviews | Item development; Pilot testing - questionnaire |
| Sandler, 2018, USA (24) | PRO-CTCAE | Various cancers - radiation to the brain, head and neck, breast, thorax, abdomen, or pelvis | Qualitative open-ended interview; quantitative (PRO-CTCAE scale) | Item development |
| Herman, 2019, UK (25) | Health-related quality of Life | Metastatic pancreatic cancer | Interviews with open-ended questions | Item development, concept elicitation |
| Riva, 2020, Italy (26) | Patient Reported Outcome for Fighting Financial Toxicity | Various cancers | Focus groups; cognitive interviews | Item development; Item reduction; Validation of the questionnaire |
| Eskildsen, 2020, Denmark (27) | Cancer Patient Empowerment Questionnaire | Various cancers | Semi-structured interviews; cognitive interviews | Item development; content validation |
| Skovlund, 2020 (28) | PRO-measures on psychological issues | Metastatic melanoma | Dialogue-based tool | PROM testing in consultations between clinicians and patients |
| Brunelli, 2020, Italy (29) | Health-related quality of Life | Various cancers | Focus groups | Item pre-development; validation via mixed method design |
| Mendez, 2022, Canada (30) | Health-related quality of Life | Head and neck squamous cell carcinoma | Questionnaires | Module development; validation |
| Janse van Rensburg, 2023, Canada (31) | Symptomatic adverse events | Various cancers | Survey, videoconference interviews | Development of term core list of symptoms |
| Sohl, 2023, USA (32) | Follow-up health-related quality of Life | Colorectal cancer | Focus groups, Semi-structured interviews in follow-up electronic tool development | Qualitative feedback on COMPASS-CP CFC that informed design elements; assessment of acceptability, appropriateness, and feasibility |
| Venderbos, 2023, The Netherlands (33) | Health-related quality of Life | Prostate cancer | Survey | Testing validity |
| Delnoij, 2023, The Netherlands (34) | Health-related quality of Life | Chronic diseases, breast cancer | Patients social media blogs | Validation of content |

PROMs: patient-reported outcome measure; PRO: patient-reported outcome; GI: gastrointestinal; CML: chronic myeloid leukaemia; cGVHD: chronic graft versus host disease; PRO-CTCAE: Patient Reported Outcomes version of the Common Terminology Criteria for Adverse Events

This table presents an overview of patient involvement in oncology PROM development and validation, including domains measured, cancer types, and methods of gathering patients’ input.

**Appendix 1**

**Literature search strategy applied to Ovid MEDLINE**

| **#** | **Query** |
| --- | --- |
| 1 | "Patient Reported Outcome Measures"/ |
| 2 | ("patient? reported outcome?" or PROM or PROMs or PROs).ti,ab,kf. |
| 3 | or/1-2 [PROs] |
| 4 | "patient participation"/ |
| 5 | (patient* adj7 (involving or involvement? or engag*)).ti,ab,kf. |
| 6 | or/4-5 [patient involvement] |
| 7 | exp neoplasms/ |
| 8 | (tumo?r? or cancer? or malignan* or metasta* or oncolog* or adenoma* or blastoma* or carcinoma* or leukemi* or lymphom* or melanoma* or sarcoma*).ti,ab,kf. |
| 9 | or/7-8 [cancer] |
| 10 | 3 and 6 and 9 [PROs AND patient involvement AND cancer] |

**Literature search strategy applied to Embase.com**

| # | **Query** |
| --- | --- |
| 1 | 'patient-reported outcome'/exp |
| 2 | ('patient$ reported outcome$' OR PROM OR PROMs OR PROs):ti,ab,kw |
| 3 | #1 OR #2 |
| 4 | 'patient engagement'/exp |
| 5 | (patient* NEAR/7 (involving OR involvement$ OR engag*)):ti,ab,kw |
| 6 | #4 OR #5 |
| 7 | 'neoplasm'/exp |
| 8 | (tumo$r$ OR cancer$ OR malignan$ OR metasta* OR oncolog* OR adenoma* OR blastoma* OR carcinoma* OR leukemi* OR lymphom* OR melanoma* OR sarcoma*):ti,ab,kw |
| 9 | #7 OR #8 |
| 10 | #3 AND #6 AND #9 |
| 11 | #10 NOT ('conference abstract'/it OR 'conference review'/it) |
| 12 | #10 AND ('conference abstract'/it OR 'conference review'/it) |

**Literature search strategy applied to Cochrane library**

| **#** | **Query** |
| --- | --- |
| 1 | [mh ^"Patient Reported Outcome Measures"] |
| 2 | ((patient* NEXT reported NEXT outcome*) OR PROM OR PROMs OR PROs):ti,ab,kw |
| 3 | #1 OR #2 |
| 4 | [mh "patient participation"] |
| 5 | (patient* NEAR/7 (involving OR involvement? OR engag*)):ti,ab,kw |
| 6 | #4 OR #5 |
| 7 | [mh neoplasms] |
| 8 | (tumo?r? OR cancer? OR malignan* OR metasta* OR oncolog* OR adenoma* OR blastoma* OR carcinoma* OR leukemi* OR lymphom* OR melanoma* OR sarcoma*):ti,ab,kw |
| 9 | #7 OR #8 |
| 10 | #3 AND #6 AND #9 |

**Literature search strategy applied to Scopus**

| **Query** |
| --- |
| (  TITLE-ABS("patient* reported outcome*" OR PROM OR PROMs OR PROs)  OR AUTHKEY("patient* reported outcome*" OR PROM OR PROMs OR PROs)  )  AND (  TITLE-ABS(patient* W/6 (involving OR involvement OR engag*))  OR AUTHKEY(patient* W/6 (involving OR involvement OR engag*))  )  AND (  TITLE-ABS(tumor OR tumour OR cancer OR malignan* OR metasta* OR oncolog* OR adenoma OR blastoma OR carcinoma OR leukemi OR lymphom OR melanoma OR sarcoma)  OR AUTHKEY(tumor OR tumour OR cancer OR malignan* OR metasta* OR oncolog* OR adenoma OR blastoma OR carcinoma OR leukemi OR lymphom OR melanoma OR sarcoma)  ) |

**References**

1. Cella DF, Tulsky DS, Gray G, Sarafian B, Linn E, Bonomi A, et al. The Functional Assessment of Cancer Therapy scale: development and validation of the general measure. Journal of Clinical Oncology. 1993 Mar;11(3):570–9.

2. Sprangers MAG, Cull A, Bjordal K, Groenvold M, Aaronson NK. The European Organization for Research and treatment of cancer approach to quality of life assessment: guidelines for developing questionnaire modules. Quality of Life Research. 1993 Aug;2(4):287–95.

3. Hearn J, Higginson IJ. Development and validation of a core outcome measure for palliative care: the palliative care outcome scale. Qual Saf Health Care. 1999 Dec 1;8(4):219–27.

4. Eisbruch A, Kim HM, Terrell JE, Marsh LH, Dawson LA, Ship JA. Xerostomia and its predictors following parotid-sparing irradiation of head-and-neck cancer. International Journal of Radiation Oncology*Biology*Physics. 2001 Jul;50(3):695–704.

5. Vickery CW, Blazeby JM, Conroy T, Arraras J, Sezer O, Koller M, et al. Development of an EORTC disease-specific quality of life module for use in patients with gastric cancer. Eur J Cancer. 2001 May;37(8):966–71.

6. Mystakidou K, Tsilika E, Kouloulias V, Parpa E, Katsouda E, Kouvaris J, et al. The “Palliative Care Quality of Life Instrument (PQLI)” in terminal cancer patients. Health Qual Life Outcomes. 2004;2(1):8.

7. Cano SJ, Browne JP, Lamping DL, Roberts AHN, McGrouther DA, Black NA. The Patient Outcomes of Surgery—Head/Neck (POS-Head/Neck): A new patient-based outcome measure. Journal of Plastic, Reconstructive & Aesthetic Surgery. 2006 Jan;59(1):65–73.

8. Rhee JS, Matthews BA, Neuburg M, Logan BR, Burzynski M, Nattinger AB. Validation of a Quality-of-Life Instrument for Patients With Nonmelanoma Skin Cancer. Arch Facial Plast Surg. 2006 Sep 1;8(5):314–8.

9. Rhee JS, Matthews BA, Neuburg M, Burzynski M, Nattinger AB. Creation of a Quality of Life Instrument for Nonmelanoma Skin Cancer Patients. Laryngoscope. 2005 Jul;115(7):1178–85.

10. Rodrigues G, Bauman G, Lock M, D’Souza D, Mahon J. Psychometric properties of a prostate cancer radiation late toxicity questionnaire. Health Qual Life Outcomes. 2007 Dec 31;5(1):29.

11. Helbostad JL, Hølen JChr, Jordhøy MS, Ringdal GI, Oldervoll L, Kaasa S. A First Step in the Development of an International Self-Report Instrument for Physical Functioning in Palliative Cancer Care: A Systematic Literature Review and an Expert Opinion Evaluation Study. J Pain Symptom Manage. 2009 Feb;37(2):196–205.

12. Wang P. Development of a patient-reported outcome instrument for chronic gastrointestinal diseases: item selection. Journal of Chinese Integrative Medicine. 2012 Oct 15;1092–8.

13. Govender R, Lee MT, Davies TC, Twinn CE, Katsoulis KL, Payten CL, et al. Development and preliminary validation of a patient-reported outcome measure for swallowing after total laryngectomy (SOAL questionnaire). Clinical Otolaryngology. 2012 Dec;37(6):452–9.

14. Wagner S, Serve H. [Digital Medicine in Oncology: Clinical Decision Support, Real World Data and Patient Involvement]. Dtsch Med Wochenschr [Internet]. 2019;144(7):430–4. Available from: http://ovidsp.ovid.com/ovidweb.cgi?T=JS&PAGE=reference&D=med16&NEWS=N&AN=30925595

15. Senn B, Mueller MD, Hasenburg A, Blankenstein T, Kammermann B, Hartmann A, et al. Development of a Postsurgical Patient-Reported Outcome Instrument for Women With Vulvar Neoplasia. Oncol Nurs Forum. 2012 Nov 1;39(6):E489–98.

16. Glaser AW, Fraser LK, Corner J, Feltbower R, Morris EJA, Hartwell G, et al. Patient-reported outcomes of cancer survivors in England 1–5 years after diagnosis: a cross-sectional survey. BMJ Open. 2013;3(4):e002317.

17. Flynn KE, Lin L, Cyranowski JM, Reeve BB, Reese JB, Jeffery DD, et al. Development of the NIH PROMIS® Sexual Function and Satisfaction Measures in Patients with Cancer. J Sex Med. 2013 Feb 1;10(Supplement_1):43–52.

18. Thomson HJ, Winters ZE, Brandberg Y, Didier F, Blazeby JM, Mills J. The early development phases of a European Organisation for Research and Treatment of Cancer (EORTC) module to assess patient reported outcomes (PROs) in women undergoing breast reconstruction. Eur J Cancer. 2013 Mar;49(5):1018–26.

19. Winters ZE, Balta V, Thomson HJ, Brandberg Y, Oberguggenberger A, Sinove Y, et al. Phase III development of the European Organization for Research and Treatment of Cancer Quality of Life Questionnaire module for women undergoing breast reconstruction. British Journal of Surgery. 2014 Feb 17;101(4):371–82.

20. Mathias SD, Chren MM, Colwell HH, Yim YM, Reyes C, Chen DM, et al. Assessing Health-Related Quality of Life for Advanced Basal Cell Carcinoma and Basal Cell Carcinoma Nevus Syndrome. JAMA Dermatol. 2014 Feb 1;150(2):169.

21. Williams LA, Agarwal S, Bodurka DC, Saleeba AK, Sun CC, Cleeland CS. Capturing the Patient’s Experience: Using Qualitative Methods to Develop a Measure of Patient-Reported Symptom Burden: An Example From Ovarian Cancer. J Pain Symptom Manage. 2013 Dec;46(6):837–45.

22. Hay JL, Atkinson TM, Reeve BB, Mitchell SA, Mendoza TR, Willis G, et al. Cognitive interviewing of the US National Cancer Institute’s Patient-Reported Outcomes version of the Common Terminology Criteria for Adverse Events (PRO-CTCAE). Quality of Life Research. 2014 Feb 20;23(1):257–69.

23. Absolom K, Gibson A, Velikova G. Engaging Patients and Clinicians in Online Reporting of Adverse Effects During Chemotherapy for Cancer: The eRAPID System (Electronic Patient Self-Reporting of Adverse Events: Patient Information and aDvice). Med Care [Internet]. 2019;57 Suppl 5 Suppl 1:S59–65. Available from: http://ovidsp.ovid.com/ovidweb.cgi?T=JS&PAGE=reference&D=med16&NEWS=N&AN=30985598

24. Sandler KA, Mitchell SA, Basch E, Raldow AC, Steinberg ML, Sharif J, et al. Content Validity of Anatomic Site-Specific Patient-Reported Outcomes Version of the Common Terminology Criteria for Adverse Events (PRO-CTCAE) Item Sets for Assessment of Acute Symptomatic Toxicities in Radiation Oncology. International Journal of Radiation Oncology*Biology*Physics. 2018 Sep;102(1):44–52.

25. Herman JM, Kitchen H, Degboe A, Aldhouse NVJ, Trigg A, Hodgin M, et al. Exploring the patient experience of locally advanced or metastatic pancreatic cancer to inform patient-reported outcomes assessment. Quality of Life Research. 2019 Nov 4;28(11):2929–39.

26. Riva S, Arenare L, Di Maio M, Efficace F, Montesarchio V, Frontini L, et al. Cross-sectional study to develop and describe psychometric characteristics of a patient-reported instrument (PROFFIT) for measuring financial toxicity of cancer within a public healthcare system. BMJ Open. 2021 Oct 20;11(10):e049128.

27. Eskildsen NB, Ross L, Bulsara C, Dietz SM, Thomsen TG, Groenvold M, et al. Development and content validation of a questionnaire measuring patient empowerment in cancer follow-up. Quality of Life Research. 2020 Aug 9;29(8):2253–74.

28. Skovlund PC, Ravn S, Seibaek L, Thaysen HV, Lomborg K, Nielsen BK. The development of PROmunication: a training-tool for clinicians using patient-reported outcomes to promote patient-centred communication in clinical cancer settings. J Patient Rep Outcomes [Internet]. 2020;4(1):10. Available from: http://ovidsp.ovid.com/ovidweb.cgi?T=JS&PAGE=reference&D=pmnm5&NEWS=N&AN=32048085

29. Brunelli C, Borreani C, Caraceni A, Roli A, Bellazzi M, Lombi L, et al. PATIENT VOICES, a project for the integration of the systematic assessment of patient reported outcomes and experiences within a comprehensive cancer center: a protocol for a mixed method feasibility study. Apolone G Belli F Borreani C Brunelli C Capri G Caraceni A Casali P Corradini P de Braud F Foschi AM Folli S Garassino M Licitra L Nicolai N Pellegrini C Platania M Procopio G Roli A Salvioni R Spada P Valdagni R Zito E BM, group PV study, editors. Health Qual Life Outcomes [Internet]. 2020;18(1):252. Available from: http://ovidsp.ovid.com/ovidweb.cgi?T=JS&PAGE=reference&D=med18&NEWS=N&AN=32723341

30. Mendez AI, Wihlidal JGJ, Eurich DT, Nichols AC, MacNeil SD, Seikaly HR. Validity of functional patient-reported outcomes in head and neck oncology: A systematic review. Oral Oncol. 2022 Feb;125:105701.

31. Janse van Rensburg HJ, Liu Z, Watson GA, Veitch ZW, Shepshelovich D, Spreafico A, et al. A tailored phase I-specific patient-reported outcome (PRO) survey to capture the patient experience of symptomatic adverse events. Br J Cancer. 2023 Sep 7;129(4):612–9.

32. Sohl SJ, Duncan PW, Thakur E, Puccinelli-Ortega N, Salsman JM, Russell G, et al. Adaptation of a Personalized Electronic Care Planning Tool for Cancer Follow-up Care: Formative Study. JMIR Form Res. 2023 Jan 10;7:e41354.

33. Venderbos LDF, Remmers S, Deschamps A, Dowling J, Carl EG, Pereira-Azevedo N, et al. The Europa Uomo Patient Reported Outcome Study 2.0-Prostate Cancer Patient-reported Outcomes to Support Treatment Decision-making. Eur Urol Focus [Internet]. 2023; Available from: http://ovidsp.ovid.com/ovidweb.cgi?T=JS&PAGE=reference&D=medp&NEWS=N&AN=37268512

34. Delnoij DMJ, Derks M, Koolen L, Shekary S, Suitela J. Using Patient Blogs on Social Media to Assess the Content Validity of Patient-Reported Outcome Measures: Qualitative Analysis of Patient-Written Blogs. JMIR Form Res [Internet]. 2023;7:e43210. Available from: http://ovidsp.ovid.com/ovidweb.cgi?T=JS&PAGE=reference&D=pmnm&NEWS=N&AN=37505797
